# Association between urinary cadmium exposure and metabolic syndrome and the mediating role of thyroid dysfunction: NHANES 2007 to 2012

**DOI:** 10.1097/MD.0000000000048230

**Published:** 2026-04-10

**Authors:** Jie Peng, Sijiong Wang, Ye Liu, Lichao Zhu, Yating Zhao

**Affiliations:** a Breast Disease Treatment Center, Affiliated Hospital of North China University of Science and Technology, Tangshan, Hebei Province, China; b The First Clinical Medical College of Gansu University of Chinese Medicine, Lanzhou, China.

**Keywords:** metabolic syndrome, NHANES, T4, thyroid function, TSH, urinary cadmium

## Abstract

This study aims to examine the association between urinary cadmium (U-Cd) exposure and metabolic syndrome (MetS) in U.S. adults and evaluate whether thyroid function (TSH/T4) mediates this relationship. We analyzed National Health and Nutrition Examination Survey 2007–2012 data (n = 4869; age ≥18) to assess the relationship between U-Cd and MetS. U-Cd was measured by ICP-MS, adjusted for creatinine, and log-transformed. We employed weighted logistic regression, restricted cubic splines for nonlinearity, and multivariable models for U-Cd–TSH/T4 relations, with causal mediation analysis for indirect effects, considering demographics and lifestyle factors. Higher U-Cd was associated with greater MetS risk in a dose–response fashion: versus Q1, OR (95% CI) for Q2, Q3, Q4 were 1.84 (1.49–2.26), 2.36 (1.92–2.89), and 2.49 (2.04–3.06); *P*-trend <.0001. Restricted cubic splines showed a significant overall and nonlinear association (*P*_overall = .006; *P*_nonlinear = .036), with relatively flat risk at low exposure and steeper increases beyond a turning point. U-Cd correlated positively with T4 (fully adjusted β ≈ 0.26 ng/dL per unit increase; *P* <.001) and inversely with TSH. Mediation indicated a small but significant indirect effect via T4 (indirect effect ≈ 0.003; 95% CI, 0.001–0.005; proportion mediated ≈ 8.3%), while TSH and TSH/T4 showed negligible or negative indirect effects; the direct path from U-Cd to MetS predominated. Findings were generally stronger in women, younger adults (<40 years), and those with higher physical activity, and were robust across chronic-disease strata. In a nationally representative sample, U-Cd exposure is positively – and nonlinearly – associated with MetS; this relationship is driven mainly by a direct effect with a modest mediating role of T4, underscoring the metabolic implications of environmental cadmium and the relevance of thyroid pathways.

## 1. Introduction

Metabolic syndrome (MetS) comprises a cluster of metabolic abnormalities – insulin resistance, obesity, hyperglycemia, hypertension, and dyslipidemia – that collectively increase the risk of cardiovascular disease, type 2 diabetes, chronic kidney disease, and other metabolic conditions.^[1–3]^ Globally, the prevalence of MetS has risen steadily in recent decades. In the United States, prevalence rose from 32.9% in 2003 to 34.7% in 2011 and reached 41.8% by 2018.^[4]^ Evidence indicates that MetS and its complications pose a growing threat to population health, particularly given the rising prevalence of overweight and obesity among children and adolescents.^[5]^ Prior studies suggest that the rising burden of MetS is driven primarily by insufficient physical activity, overnutrition, population aging, and inadequate sleep. Growing evidence also implicates environmental factors – particularly air pollution – in the onset and progression of chronic metabolic diseases.^[6]^

Rapid urbanization and industrialization have markedly increased metal pollution, including heavy metals and metalloids. Heavy metals are widely used across industrial sectors and, once released into the environment, are resistant to degradation and biodegradation.^[7]^ Recent studies examine the relationship between metal mixtures and MetS under a mixed-exposure framework and seek to identify harmful constituents within these complex mixtures. Epidemiological evidence suggests that cadmium exposure may increase the risk of MetS, although associations between metal burdens and individual MetS components are not entirely consistent.^[8]^ Conversely, some studies reported overall inverse associations – driven largely by magnesium (Mg) and molybdenum (Mo) – whereas a meta-analysis indicates that toxic metals such as arsenic, cadmium, lead, and mercury may induce MetS through shared mechanisms.^[9]^

The thyroid synthesizes and secretes thyroid hormones (THs), principally L-thyroxine (T4) and 3,5,3′-triiodo-L-thyronine (T3), which are central to metabolic and growth regulation.^[10,11]^ Thyroid dysfunction perturbs circulating TH levels, leading to hyperthyroidism or hypothyroidism. Thyroid dysfunction is considered an important contributor to MetS, and urinary cadmium (U-Cd) may promote MetS by disrupting thyroid function.^[12]^ Although existing evidence links cadmium exposure with both MetS and thyroid dysfunction, the biological mechanisms remain unclear.^[13]^ We therefore used NHANES data to examine the association between U-Cd exposure and MetS and to evaluate the potential mediating role of thyroid dysfunction. Our aim was to elucidate interrelationships among U-Cd exposure, thyroid function, and MetS, thereby informing targeted public-health interventions.

## 2. Methods

### 2.1. Study design and population

**Figure 1.**
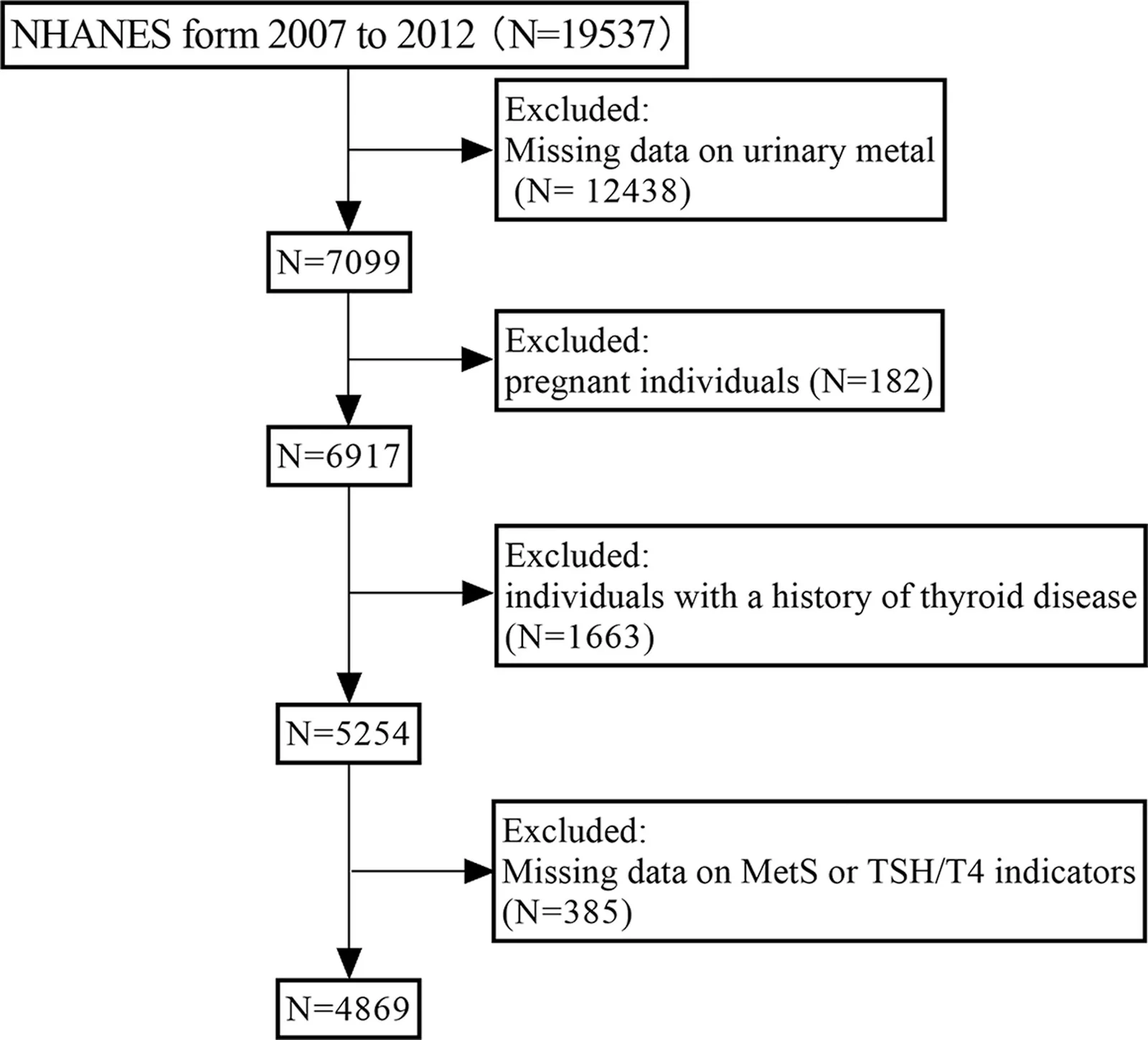
Study flow chart.

This study analyzed data from the National Health and Nutrition Examination Survey (NHANES). NHANES, administered by the National Center for Health Statistics at the U.S. Centers for Disease Control and Prevention (CDC), uses a stratified, multistage probability design to sample the non-institutionalizedU.S. population representatively. The survey integrates household interviews, standardized medical examinations, and laboratory assessments to evaluate health and nutritional status.^[14,15]^ We included 3 consecutive 2-year cycles (2007–2012). Eligible participants were aged ≥18 years and had data on U-Cd (and related urinary metals), thyroid function (TSH and T4), the 5 MetS components (waist circumference, blood pressure, fasting glucose, triglycerides, HDL-cholesterol), and core covariates (sex, age, race/ethnicity). We excluded 12,438 participants without urinary metal data, 182 pregnant individuals, 1663 with a history of thyroid disease, and 385 lacking key MetS or TSH/T4 indicators. In total, 4869 participants were included in the final analysis (Fig. 1).

### 2.2. Measurement of urinary metals and MetS

Urine samples were collected by trained staff at the Mobile Examination Center (MEC) and stored at −30°C. We quantified U-Cd using inductively coupled plasma mass spectrometry (ICP-MS). Values below the limit of detection (LOD) were imputed as LOD/√2 in accordance with NHANES protocols. To address urine dilution, metal concentrations were creatinine-standardized (µg/g creatinine) and log-transformed to improve distributional properties^.[16,17]^ MetS was defined according to the National Cholesterol Education Program Adult Treatment Panel III (NCEP ATP III): individuals meeting ≥3 of the following 5 criteria were classified as having MetS^[18]^: Central obesity (waist ≥102 cm in men, ≥88 cm in women); triglycerides ≥150 mg/dL; low HDL-cholesterol (<40 mg/dL in men, <50 mg/dL in women); hypertension (SBP ≥130 mm Hg or DBP ≥85 mm Hg, or antihypertensive use); fasting glucose ≥100 mg/dL or glucose-lowering treatment. All measurements followed standardized NHANES procedures.

### 2.3. Definition of covariates

The following covariates were included in statistical models: age (years, continuous); sex (male/female); race/ethnicity (Mexican American, other Hispanic, non-Hispanic White, non-Hispanic Black, other); educational attainment (less than high school, high school graduate, more than high school); marital status (unmarried, married); poverty-income ratio (PIR; ≤1.3, 1.3–3.5, >3.5). Body mass index (BMI, kg/m^2^; ≤25 normal weight, >25–30 overweight, ≥30 obese); physical activity (≤600 vs >600 min/week); smoking (yes/no); alcohol consumption (yes/no); sleep disorder (yes/no); hypertension (yes/no); diabetes (yes/no); MetS (yes/no); sleep duration (min/day, continuous); sedentary time (min/week, continuous); thyroid function indices, TSH and total T4.

### 2.4. Statistical analysis

We first curated the analytic dataset. Categorical variables were summarized as counts and survey-weighted percentages; between-group differences were evaluated using chi-square tests, with continuity-corrected or Rao–Scott χ^2^ tests applied for complex-survey data. Continuous variables were assessed for normality (Shapiro–Wilk) and homoscedasticity (Levene). For approximately normal and homoscedastic variables, results were reported as mean ± SD and compared using *t*-tests (two groups) or 1-way ANOVA/Wald F tests (multiple groups); when variances were unequal, Welch *t*-test or robust ANOVA was used. Given NHANES’s multistage design, all analyses incorporated survey weights following NHANES guidance. For pooling the 2007 to 2012 cycles, combined weights were computed as (1/2) × WTMEC2YR 11–12 + (1/2) × WTMEC2YR 13–14, where WTMEC2YR 11–12 and WTMEC2YR 13–14 denote cycle-specific MEC weights. Descriptive statistics reported continuous variables as mean ± SD and categorical variables as survey-weighted percentages. To assess the association between U-Cd and MetS, we fitted survey-weighted logistic regression models in 3 tiers: Model 1 (unadjusted); Model 2 (adjusted for age, sex, race/ethnicity); and Model 3 (additionally adjusted for education, PIR, marital status, smoking, alcohol consumption, BMI, and sleep duration). All analyses were performed in R 4.3.1. We report odds ratios with 95% confidence intervals, with a 2-sided α of 0.05.

## 3. Results

### 3.1. Baseline characteristics

Table 1 presents survey-weighted baseline characteristics for 4869 participants stratified by U-Cd quartiles (Q1–Q4). Overall, most characteristics differed significantly across U-Cd quartiles (many *P* <.05). Compared with Q1, participants in higher U-Cd quartiles were older (Q4 median ~52.6 years vs ~37.1 years in Q1; *P* <.001), and race/ethnicity distributions differed: the proportion of non-Hispanic Whites was lower, whereas that of non-Hispanic Blacks was higher (*P* <.001). Educational attainment declined with increasing U-Cd (the proportion with less than high school was highest in Q4; *P* <.001). The PIR also decreased with increasing U-Cd (PIR ≤1.3 most common in Q4; *P* <.001). For physical activity, the proportion achieving >600 min/week was lower at higher U-Cd levels (*P* <.001), whereas a binary “physical activity: yes” indicator was higher (*P* <.001), suggesting opposite distributions across metrics. The prevalence of smoking increased across U-Cd quartiles (highest in Q4; *P* <.001), whereas alcohol use differed statistically (*P* = .028) but with small absolute differences. BMI categories differed significantly (*P* <.001), with a greater overall proportion of BMI >25 kg/m^2^ at higher U-Cd levels. For chronic conditions, the prevalence of diabetes (*P* <.001) and MetS increased with U-Cd; sleep disorders were more frequent at higher U-Cd levels (*P* = .005); by contrast, hypertension, sex, and marital status did not differ significantly (*P* = .092, .133, and .522, respectively). Regarding thyroid function, TSH did not differ across quartiles (*P* = .318), whereas T4 increased modestly with U-Cd (*P* = .012). Sleep duration did not differ across quartiles (*P* = .510), whereas sedentary time was modestly higher at higher U-Cd levels (*P* = .034).

**Table 1:** Baseline characteristics of the study participants in NHANES.

Variable	Total (n = 4869)	Urinary cadmium				*P*
		Q1 (0.0–0.1) n = 1214	Q2 (0.1–0.2) n = 1214	Q3 (0.2–0.5) n = 1222	Q4 (0.5–6.9) n = 1218	
Age (year)	44.90 (44.00, 45.80)	37.10 (35.99, 38.20)	44.39 (43.19, 45.60)	48.54 (47.19, 49.88)	52.60 (51.33, 53.86)	<.001
Gender (%)						
Male	52.80 (51.34, 54.26)	53.14 (50.01, 56.25)	55.47 (51.38, 59.48)	53.10 (48.94, 57.23)	48.58 (44.87, 52.31)	.133
Female	47.20 (45.74, 48.66)	46.86 (43.75, 49.99)	44.53 (40.52, 48.62)	46.90 (42.77, 51.06)	51.42 (47.69, 55.13)	
Race (%)						
Mexican American	8.57 (6.66, 10.95)	8.39 (6.30, 11.09)	10.26 (7.74, 13.48)	8.23 (6.16, 10.91)	7.06 (5.04, 9.81)	<.001
Other Hispanic	6.11 (4.57, 8.11)	6.19 (4.54, 8.39)	5.94 (4.39, 8.00)	6.14 (4.27, 8.76)	6.15 (4.32, 8.68)	
Non-Hispanic White	67.02 (62.61, 71.14)	73.50 (69.45, 77.19)	66.77 (61.47, 71.68)	64.04 (58.51, 69.22)	61.43 (54.82, 67.64)	
Non-Hispanic Black	11.30 (9.30, 13.68)	6.42 (4.96, 8.28)	10.42 (8.07, 13.35)	14.52 (11.94, 17.55)	15.73 (12.36, 19.81)	
Other Races	7.01 (5.66, 8.64)	5.50 (4.28, 7.03)	6.60 (4.85, 8.93)	7.06 (5.20, 9.51)	9.63 (6.72, 13.62)	
Education level (%)						
Less than High school graduate	19.31 (17.38, 21.40)	13.06 (10.62, 15.95)	18.09 (15.30, 21.27)	19.25 (16.69, 22.10)	29.19 (25.26, 33.46)	<.001
High school graduate	22.35 (20.31, 24.53)	17.98 (15.01, 21.37)	24.32 (21.04, 27.94)	25.86 (22.45, 29.60)	21.73 (17.95, 26.06)	
Higher than High school graduate	58.34 (55.45, 61.18)	68.97 (64.82, 72.83)	57.58 (53.21, 61.84)	54.89 (50.97, 58.75)	49.08 (44.14, 54.04)	
Marital status (%)						
Married	63.40 (60.57, 66.13)	62.05 (56.50, 67.30)	63.17 (58.53, 67.58)	66.09 (61.61, 70.30)	62.38 (58.51, 66.09)	.522
Unmarried	36.60 (33.87, 39.43)	37.95 (32.70, 43.50)	36.83 (32.42, 41.47)	33.91 (29.70, 38.39)	37.62 (33.91, 41.49)	
PIR (%)						
≤1.3	22.92 (20.33, 25.73)	22.35 (17.84, 27.62)	20.10 (17.25, 23.29)	22.43 (19.02, 26.26)	27.99 (24.09, 32.27)	<.001
>1.3,≤3.5	35.58 (33.20, 38.03)	30.70 (26.77, 34.93)	37.07 (32.82, 41.54)	36.08 (32.59, 39.71)	40.18 (36.27, 44.23)	
>3.5	41.50 (38.16, 44.93)	46.95 (42.16, 51.80)	42.82 (38.25, 47.53)	41.49 (36.78, 46.36)	31.82 (27.11, 36.94)	
BMI (%)						
≤25	33.60 (31.11, 36.18)	41.01 (35.91, 46.32)	31.33 (27.79, 35.09)	29.08 (24.93, 33.61)	30.97 (27.56, 34.60)	<.001
>25,≤30	33.60 (31.11, 36.19)	30.99 (26.41, 35.99)	34.20 (30.75, 37.81)	33.97 (29.20, 39.10)	36.19 (32.31, 40.26)	
>30	32.80 (30.85, 34.82)	27.99 (24.46, 31.81)	34.48 (31.15, 37.97)	36.95 (32.95, 41.14)	32.84 (28.96, 36.98)	
PA (%)						
≤600	33.75 (32.21, 35.33)	25.44 (22.50, 28.63)	32.59 (28.82, 36.60)	37.87 (34.22, 41.67)	42.45 (38.15, 46.86)	<.001
>600	66.25 (64.67, 67.79)	74.56 (71.37, 77.50)	67.41 (63.40, 71.18)	62.13 (58.33, 65.78)	57.55 (53.14, 61.85)	
Smoking (%)						
Yes	46.25 (43.93, 48.58)	35.46 (31.03, 40.15)	41.48 (37.59, 45.49)	45.59 (41.26, 50.00)	67.21 (61.92, 72.10)	<.001
No	53.75 (51.42, 56.07)	64.54 (59.85, 68.97)	58.52 (54.51, 62.41)	54.41 (50.00, 58.74)	32.79 (27.90, 38.08)	
Drinking (%)						
Yes	79.13 (76.88, 81.22)	82.77 (79.51, 85.60)	78.25 (73.83, 82.10)	76.37 (72.89, 79.53)	78.31 (74.41, 81.76)	.028
No	20.87 (18.78, 23.12)	17.23 (14.40, 20.49)	21.75 (17.90, 26.17)	23.63 (20.47, 27.11)	21.69 (18.24, 25.59)	
Sleep disorder (%)						
Yes	7.37 (6.42, 8.45)	4.55 (3.24, 6.34)	7.79 (5.65, 10.64)	8.97 (7.13, 11.22)	9.08 (6.91, 11.84)	.005
No	92.63 (91.55, 93.58)	95.45 (93.66, 96.76)	92.21 (89.36, 94.35)	91.03 (88.78, 92.87)	90.92 (88.16, 93.09)	
Hypertension (%)						
Yes	78.49 (75.70, 81.04)	77.32 (69.02, 83.91)	73.25 (66.68, 78.93)	81.10 (76.15, 85.21)	81.89 (77.42, 85.63)	.092
No	21.51 (18.96, 24.30)	22.68 (16.09, 30.98)	26.75 (21.07, 33.32)	18.90 (14.79, 23.85)	18.11 (14.37, 22.58)	
Diabetes (%)						
Yes	9.09 (8.06, 10.24)	6.17 (4.61, 8.21)	8.77 (6.71, 11.39)	11.36 (9.56, 13.44)	11.10 (8.97, 13.67)	<.001
No	90.91 (89.76, 91.94)	93.83 (91.79, 95.39)	91.23 (88.61, 93.29)	88.64 (86.56, 90.44)	88.90 (86.33, 91.03)	
Physical activity						
Yes	21.66 (20.06, 23.36)	12.80 (10.68, 15.26)	21.22 (18.22, 24.57)	27.00 (23.63, 30.65)	28.84 (25.22, 32.75)	<.001
No	78.34 (76.64, 79.94)	87.20 (84.74, 89.32)	78.78 (75.43, 81.78)	73.00 (69.35, 76.37)	71.16 (67.25, 74.78)	
Sleep time (min/day)	6.86 (6.79, 6.93)	6.91 (6.80, 7.02)	6.81 (6.73, 6.89)	6.85 (6.67, 7.04)	6.86 (6.70, 7.02)	.510
Sedentary time (min/week)	371.47 (351.43, 391.50)	377.35 (347.13, 407.57)	344.06 (330.79, 357.32)	375.34 (351.88, 398.80)	392.48 (306.08, 478.88)	.034
TSH	1.87 (1.80, 1.95)	1.86 (1.72, 1.99)	1.90 (1.81, 1.99)	1.93 (1.73, 2.12)	1.81 (1.73, 1.89)	.318
T4	7.83 (7.75, 7.91)	7.70 (7.59, 7.81)	7.83 (7.69, 7.96)	7.87 (7.75, 8.00)	7.96 (7.86, 8.07)	.012

BMI = body mass index, NHANES = National Health and Nutrition Examination Survey, PA = physical activity, PIR = poverty-income ratio, T4 = thyroxine, TSH = thyroid-stimulating hormone.

### 3.2. Association between U-Cd and TSH/T4

**Figure 2.**
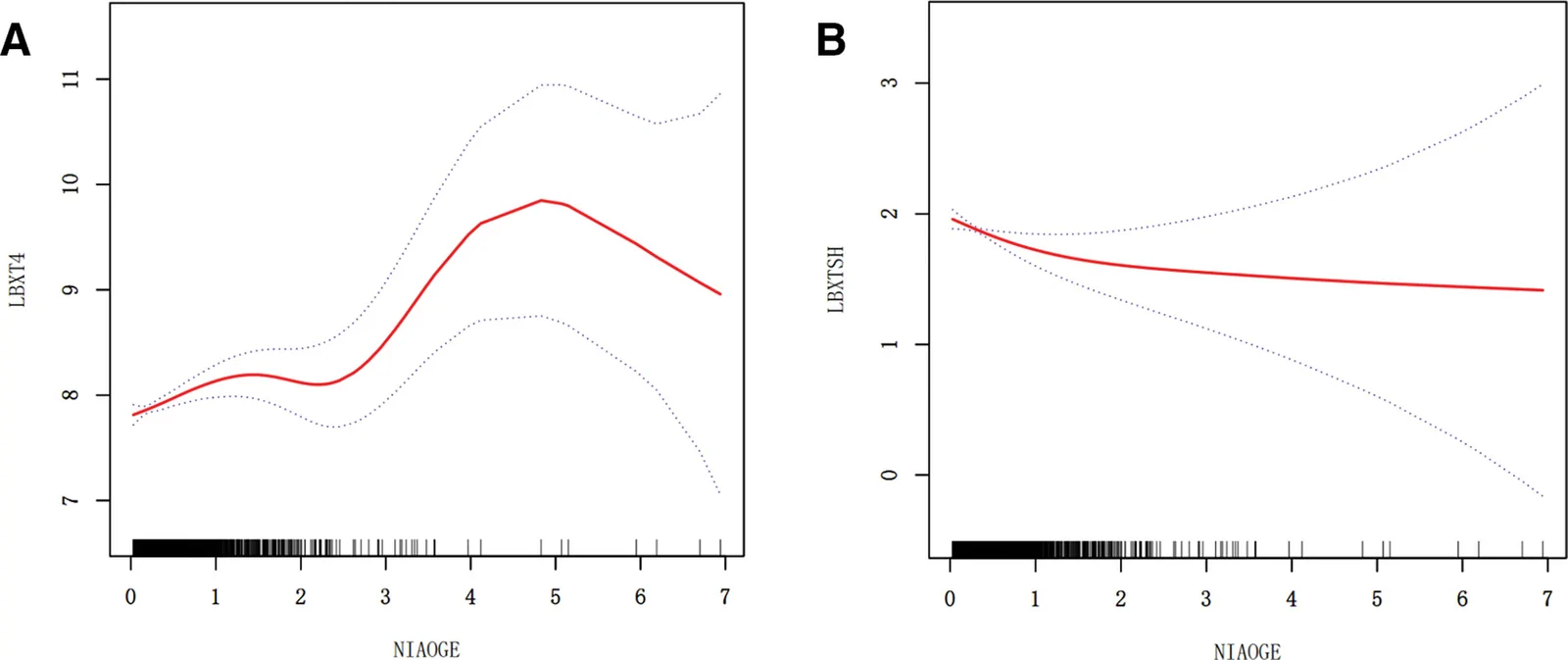
The RCS curves depict the relationship between the risk of U-Cd and TSH/T4. (A) The RCS curves depict the relationship between the risk of U-Cd and T4; (B) the RCS curves depict the relationship between the risk of U-Cd and TSH. RCS = restricted cubic spline, TSH = thyroid-stimulating hormone, U-Cd = urinary cadmium.

In multivariable linear regression, continuous U-Cd was positively associated with T4. In the unadjusted model (Model 1), each 1-unit increase in U-Cd corresponded to a 0.27 ng/dL higher T4 (95% CI, 0.18–0.36; *P* <.0001). The association persisted after adjustment for age, sex, and race/ethnicity (Model 2) and further for education, PIR, marital status, smoking, alcohol use, BMI, and sleep duration (Model 3): β = 0.26 (95% CI, 0.15–0.37; *P* <.0001). By contrast, U-Cd was inversely associated with TSH. In Model 3, each 1-unit increase in U-Cd was associated with a 0.15 µIU/mL lower TSH (95% CI, −0.29 to − 0.01; *P* = .0371). In quartile analyses, TSH in Q4 was significantly lower than in Q1 (β=−0.35; 95% CI, −0.55 to − 0.14; *P* = .0010), with a significant linear trend (*P* for trend = .0012) (Table 2). In models adjusted for age, sex, race/ethnicity, education, PIR, marital status, smoking, alcohol use, BMI, sleep duration, physical activity, and sedentary time, restricted cubic splines (RCS) indicated a nonlinear association between U-Cd and MetS (*P*_overall = .008; *P*_nonlinear = .010). When U-Cd was below 0.24 μg/L, MetS risk was slightly reduced; beyond this inflection point, higher U-Cd was associated with a marked increase in risk (Fig. 2).

**Table 2:** Weighted multivariate logistic analysis urinary cadmium and TSH/T4.

	Model 1		Model 2			Model 3		
	OR (95% CI)	*P*	OR (95% CI)		*P*		OR (95% CI)	*P*
T4								
U-Cd	0.27 (0.18–0.36)	<.001		0.23 (0.13–0.32)	<.001		0.26 (0.15–0.37)	.045
U-Cd quartile								
Q1	1.00 (Reference)	–		1.00 (Reference)	–		1.00 (Reference)	–
Q2	0.12 (−0.01 to 0.24)	.062		0.08 (−0.05 to 0.20)	.231		0.07 (−0.08 to 0.22)	.345
Q3	0.16 (0.04–0.29)	.011		0.11 (−0.01 to 0.24)	.081		0.12 (−0.03 to 0.27)	.124
Q4	0.31 (0.19–0.43)	<.001		0.23 (0.10–0.36)	<.001		0.29 (0.13–0.44)	<.001
*P* for trend	<.001	–		<.001	–		<.001	–
TSH								
U-Cd	−0.10 (−0.21 to 0.00)	.059		−0.18 (−0.29 to −0.07)	.002		−0.15 (−0.29 to −0.01)	.037
U-Cd quartile								
Q1	1.00 (Reference)	–		1.00 (Reference)	–		1.00 (Reference)	–
Q2	−0.04 (−0.19 to 0.10)	.580		−0.12 (−0.26 to 0.03)	.121		−0.12 (−0.31 to 0.08)	.238
Q3	−0.09 (−0.24 to 0.05)	.220		−0.20 (−0.35 to −0.05)	.008		−0.23 (−0.43 to −0.04)	.020
Q4	−0.15 (−0.29 to −0.00)	.047		−0.32 (−0.48 to −0.17)	<.001		−0.35 (−0.55 to −0.14)	.001
*P* for trend	.041	–		<.001	–		.001	–

The association between the urinary cadmium and TSH/T4 among all participants in the NHANES cohort. Model 1 was an unadjusted crude model; Model 2 was built upon Model 1 by incorporating adjustments for age, gender, race. Model 3 further extended Model 2 by incorporating adjustments for the PIR, marital status, smoking, drinking, BMI, sleep time.

BMI = body mass index, NHANES = National Health and Nutrition Examination Survey, PIR = poverty-income ratio, T4 = thyroxine, TSH = thyroid-stimulating hormone, U-Cd = urinary cadmium.

### 3.3. Association between U-Cd and MetS risk

**Figure 3.**
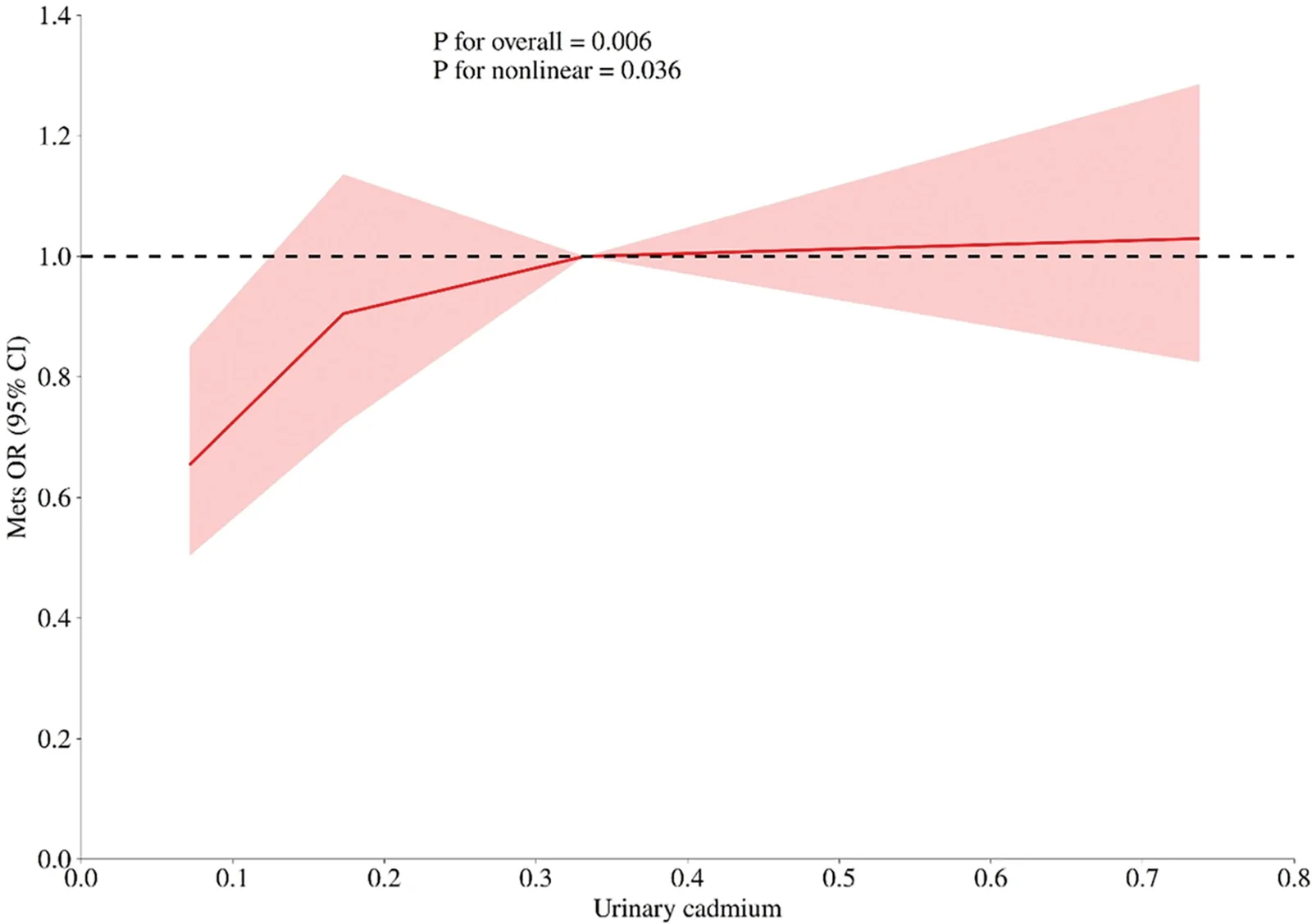
The RCS curves depict the relationship between the risk of U-Cd and Mets. RCS = restricted cubic spline, U-Cd = urinary cadmium.

Survey-weighted logistic regression across U-Cd quartiles showed a significant dose–response relationship between U-Cd and MetS risk. Relative to Q1, Q4 was associated with a higher risk of MetS (OR = 2.49; 95% CI, 2.04–3.06; *P*_trend <.0001). After multivariable adjustment (age, sex, race/ethnicity, education, PIR, marital status, smoking, alcohol use, BMI, sleep duration, physical activity, sedentary time), risks were also elevated for intermediate quartiles: Q2, OR = 1.84 (95% CI, 1.49–2.26; *P* <.0001); Q3, OR = 2.36 (95% CI, 1.92–2.89; *P* <.0001) (Table 3). In RCS models with the same covariate adjustment, the U-Cd–MetS association was overall significant and nonlinear (*P*_overall = .006; *P*_nonlinear = .036), indicating relatively larger risk changes at low exposure and a decelerating increase beyond the inflection point (Fig. 3).

**Table 3:** Weighted multivariate logistic analysis urinary cadmium and MetS.

	Model 1		Model 2		Model 3		
	OR (95% CI)	*P*	OR (95% CI)	*P*		OR (95% CI)	*P*
U-Cd	1.41 (1.24–1.60)	<.001	1.06 (0.93–1.22)	.392		1.16 (0.97–1.38)	.105
U-Cd quartile							
Q1	1.00 (Reference)	–	1.00 (Reference)	–		1.00 (Reference)	–
Q2	1.84 (1.49–2.26)	<.001	1.39 (1.12–1.73)	.003		1.39 (1.06–1.83)	.019
Q3	2.36 (1.92–2.89)	<.001	1.57 (1.27–1.95)	<.001		1.47 (1.12–1.93)	.005
Q4	2.49 (2.04–3.06)	<.001	1.38 (1.11–1.73)	.004		1.53 (1.15–2.02)	.003
*P* for trend	<.001	–	.114	–		.030	–

The association between the urinary cadmium and MetS among all participants in the NHANES cohort. Model 1 was an unadjusted crude model; Model 2 was built upon Model 1 by incorporating adjustments for age, gender, race. Model 3 further extended Model 2 by incorporating adjustments for the PIR, marital status, smoking, drinking, BMI, sleep time, physical activity, sedentary time.

MetS = metabolic syndrome, NHANES = National Health and Nutrition Examination Survey, PIR = poverty-income ratio, U-Cd = urinary cadmium.

### 3.4. Mediation analysis

**Figure 4.**
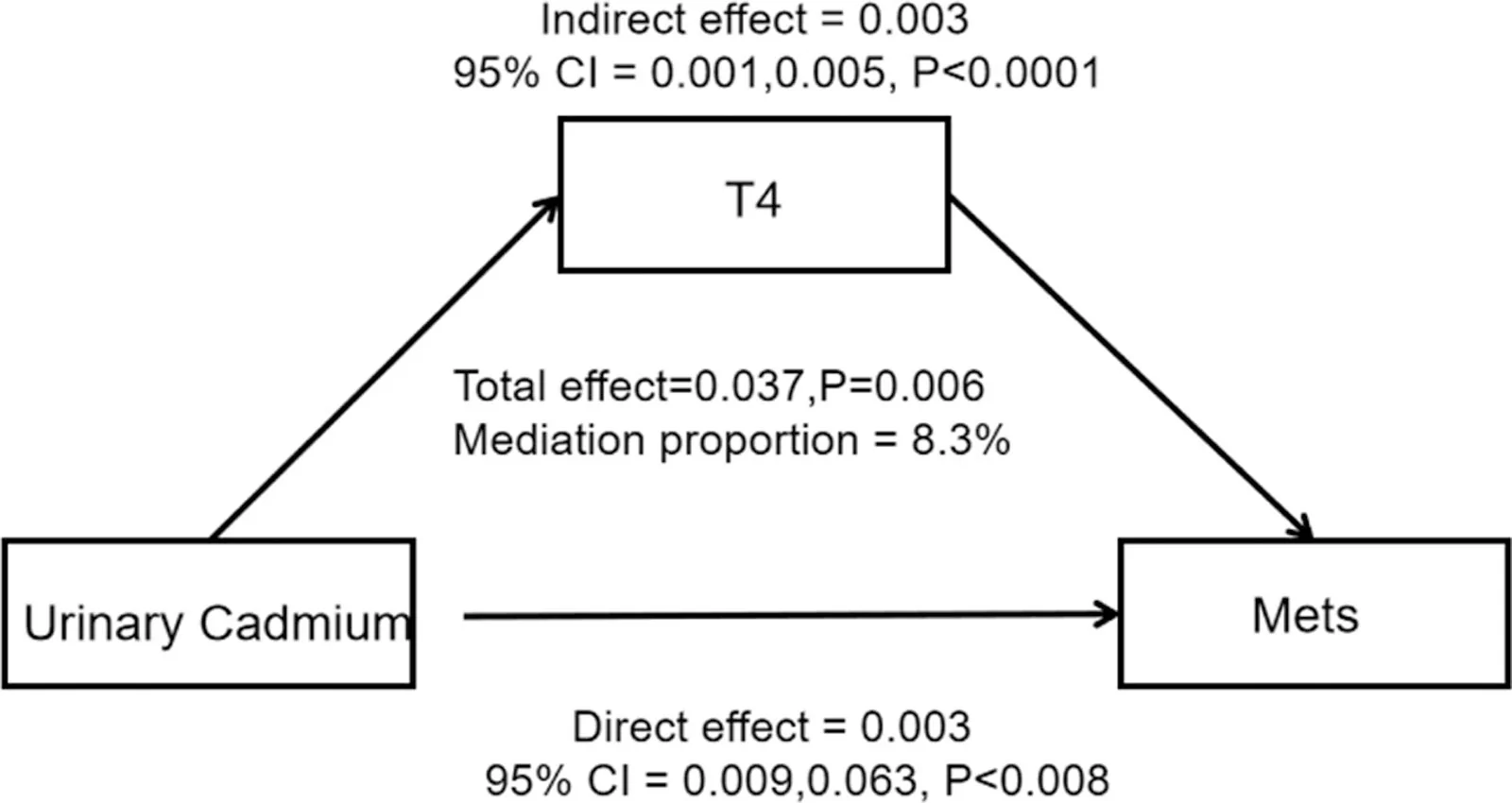
Mediation analysis model.

Based on Tables 4 and 5, after full covariate adjustment, T4 exhibited a significant positive mediating effect on the U-Cd→MetS pathway (indirect effect = 0.003; 95% CI, 0.001–0.005; *P* <.001), accounting for 8.3% of the total effect. By contrast, indirect effects via TSH and TSH/T4 were small and negative (−3.8% and − 2.1%), suggesting no positive mediation and potential suppression (Table 4). In the jointly adjusted mediation model, the overall indirect effect was not significant (estimate = 0.0005; 95% CI, −0.0015 to 0.0032; *P* = .484; proportion mediated = 0.884%), whereas the direct effect remained significant (0.0527; 95% CI, 0.0224–0.0835; *P* <.001), yielding a total effect of 0.0531 (95% CI, 0.0229–0.0841; *P* <.001). These findings indicate that the U-Cd–MetS association is driven primarily by the direct pathway, with T4 providing a limited yet significant mediating contribution, whereas pathways via TSH or the TSH/T4 ratio are not supported (Table 5 and Fig. 4).

**Table 4:** Mediation analysis of TSH, T4, and the TSH/T4 ratio in the association between urinary cadmium and metabolic syndrome.

Mediation effect (95% CI), value							
	Total effect	*P*	Indirect effect	*P*	Direct effect	*P*	Mediation
T4	0.037 (0.012–0.066)	.006	0.003 (0.001 to 0.005)	<.001	0.034 (0.009–0.063)	.008	8.3%
TSH	0.036 (0.012–0.066)	.006	−0.001 (−0.004 to −0.0001)	0.04	0.038 (0.013–0.067)	.006	−3.8%
TSH/T4	0.037 (0.011–0.065)	.006	−0.001 (−0.004 to 0.001)	0.298	0.037 (0.012–0.066)	.006	−2.1%

T4 = thyroxine, TSH = thyroid-stimulating hormone.

**Table 5:** Covariate-adjusted mediation analysis for the urinary cadmium–metabolic syndrome association.

Effect	Estimate	Lower	Upper	β (95% CI)	*P*	Mediation %
Indirect	0.0005	−0.0015	0.0032	0.0005 (−0.0015 to 0.0032)	.484	0.884
Direct	0.0527	0.0224	0.0835	0.0527 (0.0224–0.0835)	<.001	99.117
Total	0.0531	0.0229	0.0841	0.0531 (0.0229–0.0841)	<.001	100.000

This table shows results adjusted for age, sex, race/ethnicity, education, poverty-income ratio, marital status, smoking, alcohol consumption, body mass index, sleep duration, physical activity, and sedentary time.

### 3.5. Subgroup analyses

**Figure 5.**
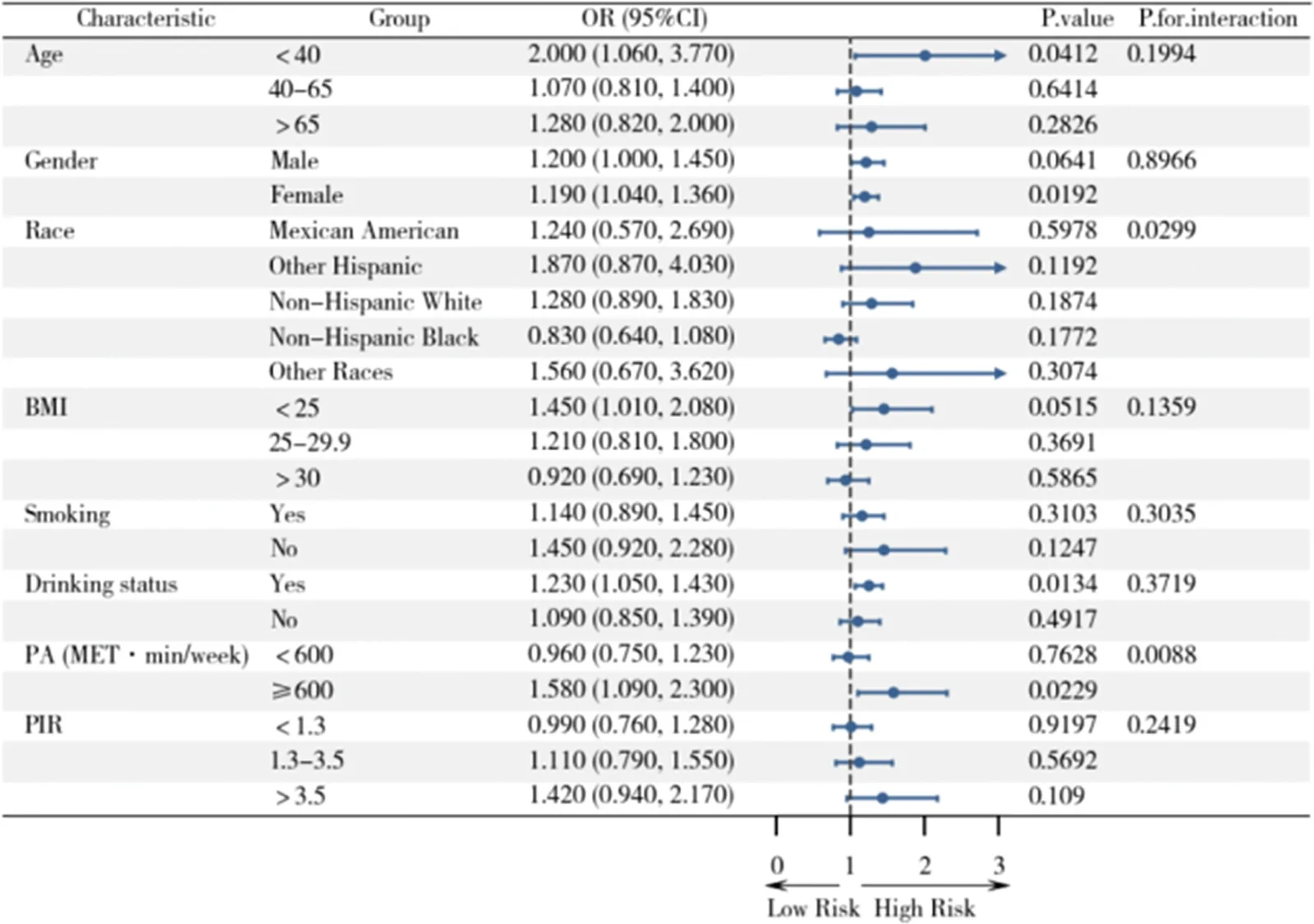
Subgroup analyses.

**Figure 6.**
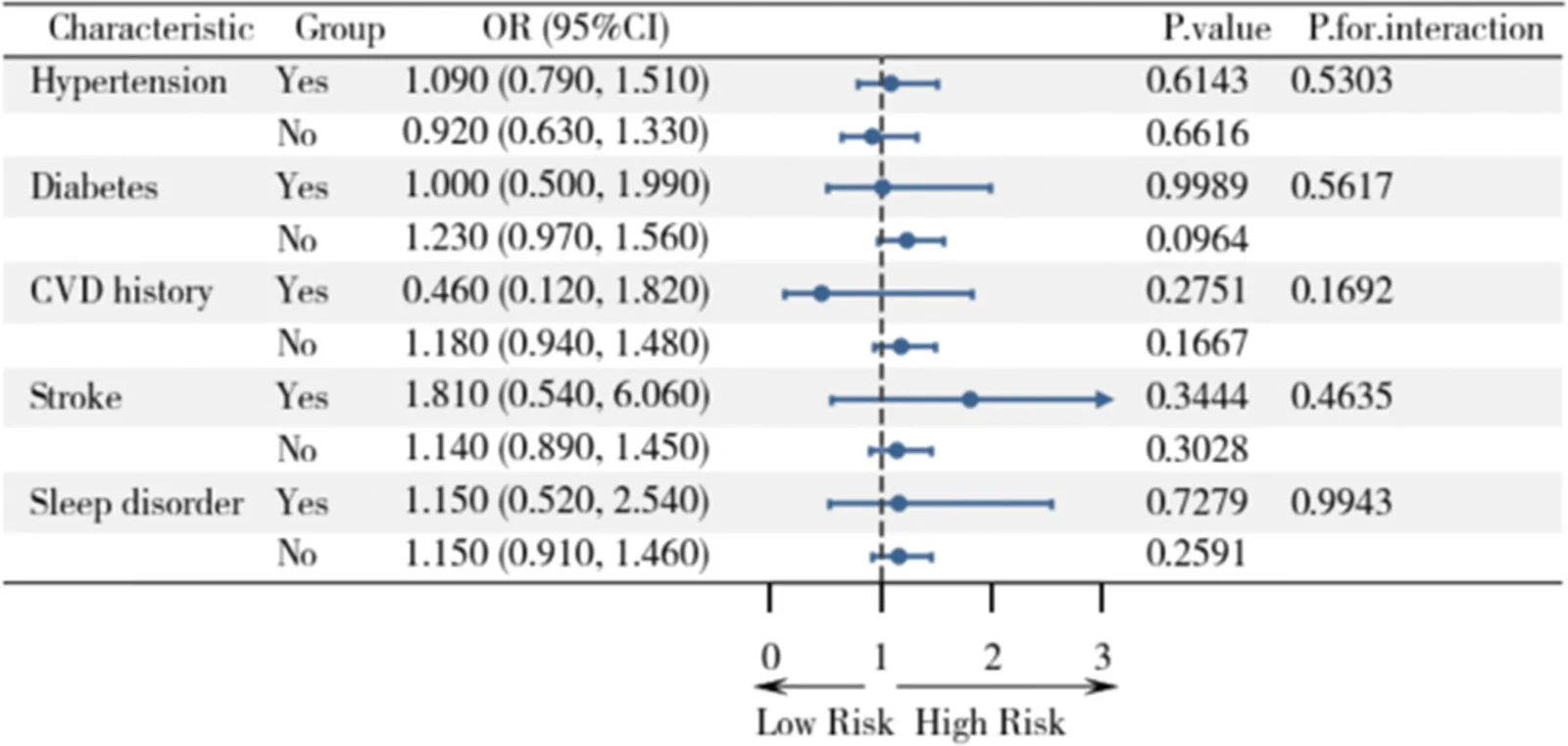
Subgroup analyses by chronic conditions.

After adjustment for age, sex, race/ethnicity, education, BMI, smoking, alcohol use, diabetes, sleep duration, physical activity, and sedentary time, several subgroups showed stronger associations. Among adults <40 years, the U-Cd–MetS association was significant (OR = 2.00; 95% CI, 1.06–3.77; *P* = .041), although the age interaction was not (*P*_interaction = .199). The association was significant in women (OR = 1.19; 95% CI, 1.04–1.36; *P* = .019) but not in men (OR = 1.20; 95% CI, 1.00–1.45; *P* = .064), and the sex interaction was not significant (*P*_interaction = .897). Physical activity demonstrated effect modification: participants with ≥600 MET-min/week showed a stronger association (OR = 1.58; 95% CI, 1.09–2.30; *P* = .023), whereas those with <600 did not (OR = 0.96; *P* = .763). The interaction was significant (*P*_interaction = .009), suggesting that physical activity may modify the association. An overall race/ethnicity interaction was observed (*P*_interaction = .030), although no individual subgroup reached statistical significance. Other stratifications (BMI, smoking, alcohol use, PIR) provided no consistent evidence of interaction; among drinkers, the association was significant (OR = 1.23; *P* = .013), but the interaction with nondrinkers was not (*P*_interaction = .372) (Table 6 and Fig. 5). To assess robustness, we further stratified by chronic conditions (hypertension, diabetes, prior CVD, stroke, sleep disorder). Directions were broadly consistent across subgroups, and interaction terms were not significant (all *P*_interaction >.16), indicating that the overall U-Cd–MetS association was insensitive to chronic-disease status (e.g., hypertension: OR = 1.09 vs 0.92; diabetes: OR = 1.00 vs 1.23; both not significant) (Table 7 and Fig. 6).

**Table 6:** Subgroup analysis.

Characteristic	Group	OR (95% CI)	*P*	*P* for interaction
Age	<40	2.00 (1.06–3.77)	.041	.199
	40–65	1.07 (0.81–1.40)	.641	–
	>65	1.28 (0.82–2.00)	.283	–
Gender	Male	1.20 (1.00–1.45)	.064	.897
	Female	1.19 (1.04–1.36)	.019	–
Race	Mexican American	1.24 (0.57–2.69)	.599	.030
	Other Hispanic	1.87 (0.87–4.03)	.119	–
	Non-Hispanic White	1.28 (0.89–1.83)	.187	–
	Non-Hispanic Black	0.83 (0.64–1.08)	.177	–
	Other Races	1.56 (0.67–3.62)	.307	–
BMI	<25	1.45 (1.01–2.08)	.052	.136
	25–29.9	1.21 (0.81–1.80)	.369	–
	>30	0.92 (0.69–1.23)	.587	–
Smoking	Yes	1.14 (0.89–1.45)	.310	.304
	No	1.45 (0.92–2.28)	.125	–
Drinking status	Yes	1.23 (1.05–1.43)	.013	.372
	No	1.09 (0.85–1.39)	.492	–
PA	<600	0.96 (0.75–1.23)	.763	.009
	≥600	1.58 (1.09–2.30)	.023	–
PIR	<1.3	0.99 (0.76–1.28)	.918	.24
	1.3–3.5	1.11 (0.79–1.55)	.569	–
	>3.5	1.42 (0.94–2.17)	.109	–

BMI = body mass index, PA = physical activity, PIR = poverty-income ratio.

**Table 7:** Subgroup analysis by chronic disease.

Characteristic	Group	OR (95% CI)	*P*	*P* for interaction
Hypertension	Yes	1.09 (0.79–1.51)	.614	.530
	No	0.92 (0.63–1.33)	.662	–
Diabetes	Yes	1.00 (0.50–1.99)	.999	.562
	No	1.23 (0.97–1.56)	.096	–
CVD history	Yes	0.46 (0.12–1.82)	.275	.169
	No	1.18 (0.94–1.48)	.167	–
Stroke	Yes	1.81 (0.54–6.06)	.344	.464
	No	1.14 (0.89–1.45)	.303	–
Sleep disorder	Yes	1.15 (0.52–2.54)	.728	.994
	No	1.15 (0.91–1.46)	.259	–

CVD = cardiovascular disease.

## 4. Discussion

This study provides new population-level evidence for the association between U-Cd exposure and MetS, including its component traits. Using nationally representative NHANES data, we observed a significant positive association between U-Cd exposure and the risk of MetS, suggesting that cadmium – a widespread environmental contaminant – may adversely affect metabolic health. Consistent with our findings, a case–control study from Iran^[8]^ and cross-sectional studies from China^[19]^ and Korea^[20]^ reported positive associations between U-Cd and MetS. However, 1 study suggested a potential inverse association between U-Cd and MetS, likely reflecting differences in study populations and the biological matrices used for exposure assessment^[21]^。These discrepancies may stem from differences in population structure, exposure assessment (blood vs urine, biomarker half-life and exposure window), covariate adjustment, and statistical modeling. Further analyses indicated that U-Cd was associated not only with MetS overall but also with several components (abdominal obesity, dysglycemia, and dyslipidemia), consistent with prior reports implicating cadmium in metabolic dysregulation. Notably, restricted cubic spline analyses revealed nonlinearity: risk changed little at low exposure but rose more steeply beyond a threshold, a “threshold–acceleration” pattern that can inform exposure-stratified risk management.

Regarding mechanisms, mediation analysis suggested a role for the thyroid axis in the U-Cd→MetS pathway: T4 conferred a significant positive mediating effect, whereas indirect effects via TSH and the TSH/T4 ratio were small or negative, consistent with suppression rather than positive mediation. NHANES-based studies have linked blood or U-Cd to lower TSH levels,^[22]^ and this association has been observed in both adults and adolescents.^[23]^ Three studies examining blood cadmium and thyroid function reported that higher blood cadmium was associated with an increased risk of hypothyroidism.^[24–26]^ Conversely, a study of occupational exposure reported a positive association between U-Cd and TSH,^[27]^ underscoring the importance of exposure level, temporal window, and population differences. Biologically, cadmium may alter peripheral T4/T3 levels and tissue sensitivity by inducing oxidative stress and inflammation, disrupting thyroid hormone synthesis and secretion, modulating deiodinase (DIO1/DIO2) activity, and perturbing hypothalamic–pituitary–thyroid feedback; THs centrally regulate basal metabolic rate, glucose homeostasis, and lipid metabolism.^[28,29]^ Prior work indicates that higher fT3 is linked to greater adiposity and multiple metabolic abnormalities; a higher fT3/fT4 ratio has been associated with adverse metabolic profiles and aberrant placental growth during pregnancy.^[30]^ Individuals with obesity or unfavorable metabolic status may exhibit increased DIO1/DIO2 activity, thereby enhancing the conversion of fT4 to fT3.^[31]^ This regulatory process reflects altered tissue sensitivity to THs and may affect basal metabolism, glucose uptake and utilization, and lipid handling, thereby contributing to fluctuations in glycemia and lipidemia.^[32]^ Elevated fT3 may also coincide with greater insulin resistance and impaired insulin signaling, accompanied by heightened cardiovascular activity – hallmarks of MetS. Taken together with our findings, U-Cd exposure may contribute to MetS partly through alterations in thyroid function, underscoring the endocrine impact of environmental cadmium – particularly in aging populations. Interventions targeting thyroid dysfunction may help mitigate cadmium-related risk of MetS. Future studies should delineate the longitudinal dynamics of U-Cd exposure and thyroid function and their combined impact on metabolic health, thereby informing public-health policy.

This study has several strengths. First, we leveraged a nationally representative, complex survey of the non-institutionalized U.S. population, supporting generalizability. Second, we adhered to NHANES complex-survey weighting and performed multiple model adjustments and sensitivity analyses. Third, by combining quartile-based and continuous (log/doubling) models with RCS and mediation analyses, we strengthened inference regarding both exposure–response shape and putative pathways. Nonetheless, several limitations warrant consideration. First, the cross-sectional design precludes firm temporal ordering, and reverse causation cannot be excluded. Second, our analysis was restricted to NHANES 2007–2012, which are the most recent NHANES cycles with concurrent measurements of urinary cadmium and a comprehensive thyroid profile (including TSH and total T4); therefore, the findings may not fully reflect more recent exposure patterns or changes in industrial emissions and regulation. Third, U-Cd reflects short- to intermediate-term exposure, and single or infrequent measurements are susceptible to dilution and diurnal variation, although creatinine standardization and LOD imputation were applied. Fourth, residual and unmeasured confounding may persist (e.g., dietary trace elements, iodine status, thyroid autoantibodies, occupational or geographic exposures). Fifth, some subgroups had limited sample sizes, yielding imprecise interaction estimates.

## 5. Conclusion

In conclusion, U-Cd exposure is positively associated with the risk of MetS in a dose–response and nonlinear manner; the relationship appears to be driven primarily by a direct pathway, with a modest yet significant mediating contribution via T4, and remains broadly robust across subgroups – showing stronger associations in women, younger adults, and highly active individuals.

## Author contributions

**Methodology:** Lichao Zhu.

**Software:** Sijiong Wang.

**Supervision:** Yating Zhao.

**Writing – original draft:** Jie Peng.

**Writing – review & editing:** Ye Liu.
